# Occupational Stress, Burnout, and Fatigue Among Healthcare Workers in Shanghai, China: A Questionnaire-Based Cross-Sectional Survey

**DOI:** 10.3390/healthcare13131600

**Published:** 2025-07-03

**Authors:** Qiaochu Wang, Jiayun Ding, Yiming Dai, Sijia Yang, Zhijun Zhou

**Affiliations:** 1 Key Laboratory of Public Health Safety of Ministry of Education, School of Public Health, Fudan University, No. 130 Dong’an Road, Shanghai 200032, China; 21111020010@m.fudan.edu.cn (Q.W.); 22211020077@m.fudan.edu.cn (J.D.); 20111020031@fudan.edu.cn (Y.D.); 2 Key Laboratory of Health Technology Assessment of National Health Commission, School of Public Health, Fudan University, No. 138 Yixueyuan Road, Shanghai 200032, China; 3 Shanghai Municipal Center for Disease Control and Prevention, No. 1399 Shen Hong Road, Minhang District, Shanghai 200336, China

**Keywords:** healthcare workers, occupational stress, occupational burnout, fatigue

## Abstract

**Background**: Occupational burnout and fatigue are critical issues affecting the health and performance of healthcare workers (HCWs) globally. These outcomes are often driven by complex and overlapping work-related stressors, which remain insufficiently understood in combination. **Objective**: To investigate the associations of multiple work-related stressors with occupational burnout and fatigue, and to identify distinct stress patterns and critical stressors among HCWs. **Method**: A cross-sectional survey was conducted using a self-administered electronic questionnaire among 2695 HCWs in Shanghai, China. Validated questionnaire scales were used to assess work-related stress (self-developed occupational stress scale for medical staff, CSSM), occupational burnout (Maslach Burnout Inventory–General Survey, MBI-GS), and fatigue (Fatigue Scale-14, FS-14). Latent profile analysis (LPA) was employed to identify distinct work-related stress patterns. Generalized linear models (GLMs) were used to explore the associations between individual stressors, stress patterns, and occupational burnout and fatigue. Additionally, weighted quantile sum (WQS) models were utilized to evaluate the combined effects of multiple stressors and identify the main contributors. **Results**: In this study, 77.0% and 71.2% of participants were classified as experiencing occupational burnout and fatigue, respectively. A strained doctor–patient relationship was the highest-rated work-related stressor. All work-related stressors, including career development, interpersonal relationships, work–life imbalance, physical environment, doctor–patient relationship, social environment, and workload, were significantly associated with burnout (β: 0.444~0.956, *p* < 0.001) and fatigue (β: 1.384~3.404, *p* < 0.001). WQS assigned higher weights to career development and workload for burnout, and to workload and work–life imbalance for fatigue. LPA identified two distinct occupational stress patterns. HCWs characterized by higher stress levels in physical environment, career development, workload, and interpersonal relationships exhibited significantly higher burnout scores (β = 0.325, 95% CI: 0.122, 0.528), particularly in the reduced personal accomplishment (PA) dimension (β = 1.003, 95% CI: 0.746, 1.259). **Conclusions**: This study highlighted the high prevalence of occupational burnout and fatigue among HCWs in Shanghai, China. Occupational stressors were associated with both burnout and fatigue, with higher workload, work–life imbalance, and poorer career development showing particularly significant contributions. These findings emphasized the urgent need for targeted interventions, including workload management, career development programs, and mental health support, to reduce occupational stress and mitigate its adverse effects on HCWs.

## 1. Introduction

Healthcare workers (HCWs) are an essential part of the healthcare system, and their well-being is crucial for ensuring the delivery of high-quality medical services and maintaining public health. However, the demanding nature of their profession exposes them to significant occupational challenges, such as heavy workloads, high-risk responsibilities, irregular working hours, and strained doctor–patient relationships, all of which exacerbate their occupational stress and increase their vulnerability to burnout and fatigue [1,2]. These multifaceted challenges persistently escalate in modern healthcare environments, placing sustained pressures on HCWs. Beyond individual consequences, these issues impact the broader healthcare system, leading to suboptimal patient outcomes, diminished professionalism, and an increased risk of workplace injuries [3,4,5].

Work-related stress stems from organizational factors, as well as a mismatch between job demands, individual capabilities, and the support available in the workplace [6,7]. Given the inherently high-intensity, high-risk, and high-responsibility nature of medical practice, occupational stress is particularly pronounced in the healthcare sector [8]. When such stress persists without adequate mitigation, it accumulates progressively, leading to burnout [9]. Occupational burnout is a complex psychological syndrome typically characterized by three dimensions: emotional exhaustion (EE), depersonalization (DP), and a diminished sense of personal accomplishment (PA) [9]. Fatigue, another common concern associated with chronic stress, manifests as both physical and psychological exhaustion [10,11]. These two outcomes are interconnected, with sustained stress and fatigue exacerbating the likelihood of developing burnout [12], creating a cycle that perpetuates poor mental and physical health among HCWs, ultimately impairing the functioning of the healthcare system.

Previous studies have emphasized the contribution of specific work-related factors to burnout and fatigue among HCWs [13,14,15,16]. For instance, Zhou et al. conducted a meta-analysis of studies from multiple countries and demonstrated that excessive workload, concerns about patient care, unsupportive work environments, and poor work–life balance were major occupational stressors associated with burnout among physicians [14]. Similarly, studies reported that psychological workload was a determinant of occupational fatigue among Iranian hospital service personnel, while workplace violence and a lack of organizational support were linked to chronic fatigue in Chinese nurses [15,16]. These studies mainly examined individual stressors separately; however, HCWs in real-world clinical settings are commonly exposed to multiple concurrent sources of stress. Therefore, a more comprehensive understanding of how occupational stress patterns contribute to burnout and fatigue is still needed. Additionally, identifying the most critical work-related stressors can help in developing targeted interventions to alleviate these challenges.

We conducted a cross-sectional survey among 2695 HCWs to investigate the associations between multiple work-related stressors and occupational burnout and fatigue, which were assessed using the self-developed occupational stress scale for medical staff (CSSM), the Maslach Burnout Inventory–General Survey (MBI-GS), and the Fatigue Scale-14 (FS-14), respectively. Addressing a key gap in existing research—which often focuses on isolated stressors—we employed latent profile analysis (LPA) and weighted quantile sum (WQS) regression to uncover distinct patterns of occupational stress and to identify the most influential stressors for HCWs. By capturing the complex, multifactorial nature of workplace stress, our findings offer novel insights that can guide the design of targeted, evidence-based interventions to promote HCWs’ mental well-being and optimize the quality of healthcare services.

## 2. Materials and Methods

### 2.1. Study Design and Data Collection

This study was a questionnaire-based cross-sectional survey conducted among HCWs in Shanghai, China, in September 2024 using a two-stage hybrid sampling approach combining stratified random sampling and convenience sampling. Public hospitals in China were categorized into primary (Level 1), secondary (Level 2), and tertiary (Level 3) hospitals based on their functions, facilities, and technical expertise [17]. In the first stage, five primary hospitals, three secondary hospitals, and three tertiary hospitals in Shanghai were randomly selected from each stratum to ensure proportional representation of hospital levels. In the second stage, participants were recruited through convenience sampling within the selected hospitals. The inclusion criteria were as follows: (1) HCWs from clinical departments, and (2) willingness to participate in this survey. Exclusion criteria included (1) staff from administrative or support services departments, (2) individuals with a history of mental illness or a family history of mental illness, (3) individuals with a recent history of major surgery, and (4) pregnant or breastfeeding women. The required sample size was calculated using the following formula:

$$n=\frac{z^{2}\cdot p\cdot (1-p)}{d^{2}}$$

Previous studies indicated that the proportion of Chinese HCWs with high occupational stress exceeded 60% (*p* = 0.60) [18]. Using a 95% confidence interval (*z* = 1.96) and a margin of error no greater than 2.5% (*d* = 0.025), the minimum required sample size was determined to be 1476. To enhance the representativeness of the study and account for potential non-response bias, we invited 2701 eligible HCWs to complete the questionnaire created on an online survey platform (in Chinese, “Wenjuanxing”). To reduce respondent burden and minimize survey fatigue, each participant was assigned to complete either the MBI-GS or the FS-14, but not both. The study aim was clearly stated at the beginning of the questionnaire. By filling out and completing the questionnaire, participants provided their digital consent to take part in the study. Invalid questionnaires were excluded based on the following criteria: (1) a response time of less than 120 s and (2) a high option repetition rate (>70%). Ultimately, valid data from 2695 HCWs were included in the final analysis, with an effective response rate of 99.8%. Among them, 732 HCWs completed the fatigue scale, and 1963 completed the burnout scale. This study was reviewed and approved by the Ethical Review Committee of the Shanghai Municipal Center for Disease Control and Prevention (Approval No. 2024-67).

### 2.2. Questionnaires

#### 2.2.1. General Characteristics Questionnaire

Participants’ general characteristics were collected with self-designed questionnaires, including sociodemographic information (age, gender, education level, marital status, and monthly income), work-related features (hospital classification, department, job position, work year, professional title, work time, and night shifts), and lifestyle factors (physical activity, smoking, and alcohol consumption).

#### 2.2.2. Self-Developed Occupational Stress Scale for Medical Staff (CSSM)

Currently, no specific scale exists for quantifying occupational stress among HCWs; instead, general tools such as the Job Content Questionnaire (JCQ) [19] and Effort–Reward Imbalance (ERI) [20] are commonly used. However, these scales have broad evaluation criteria and do not account for the unique stressors faced by medical staff. To address this gap, we developed the CSSM scale in our previous study [21] with reference to several well-established occupational stress assessment tools, both domestic and international, including the JCQ [19], ERI [20], HSE Management Standards Indicator Tool [22], and the Nursing Stress Scale (NSS) [23]. The CSSM was specifically designed to measure occupational stress among HCWs, consisting of 32 items across 7 dimensions: career development, interpersonal relationships, work–life imbalance, physical environment, doctor–patient relationship, social environment, and workload. Each item is rated on a 5-point Likert scale (1 = “strongly disagree” to 5 = “strongly agree”). The total stress score is the average of all item scores, while dimension scores are calculated as the mean of item scores within each dimension. Higher scores indicate greater stress levels. The scale’s reliability and validity were confirmed among HCWs in Shanghai, with significant correlations observed between the CSSM subscales and JCQ scores (r = 0.312~0.754, *p* < 0.05), and Cronbach’s α coefficients ranging from 0.743 to 0.937 [21]. The scale items along with their psychometric properties are presented in Table S1. In this study, the Cronbach’s α for the overall CSSM scale was 0.935, with subscale values ranging from 0.756 to 0.902.

#### 2.2.3. Maslach Burnout Inventory–General Survey (MBI-GS)

Occupational burnout of the participants was evaluated using the MBI-GS, a widely used instrument originally developed by Maslach and Jackson [9]. We employed its Chinese modified version culturally adapted by Li et al. [24]. This scale includes three dimensions, EE (5 items), DP (4 items), and PA (6 items), with a total of 15 items. EE refers to the loss of enthusiasm for work; DP refers to deliberately maintaining distance from work and individuals, displaying indifference and neglect toward work objects and environments; and reduced PA reflects a negative self-evaluation and a devaluation of the meaning and value of work. The questionnaire uses a 7-point Likert scale, with scores ranging from 0 (“never”) to 6 (“very frequently”). The score for each dimension is the average of the item scores within that dimension. To facilitate presentation, the PA dimension was reverse-scored by subtracting the original dimension score from 6. The total score was then calculated as (0.4 × EE + 0.3 × DP + 0.3 × PA), where higher scores indicate greater levels of burnout. Total scores below 1.5 indicate no occupational burnout, scores between 1.5 and 3.4 indicate mild to moderate burnout, and scores of 3.5 or higher indicate severe burnout. In this study, the Cronbach’s α coefficient for the MBI-GS scale was 0.885.

#### 2.2.4. Fatigue Scale-14 (FS-14)

In this study, fatigue among HCWs was assessed using the FS-14 scale [25], the Chinese version of which has demonstrated satisfactory reliability and validity in previous studies [11,26]. The FS-14 consists of two dimensions with a total of 14 items: 8 items for physical fatigue and 6 items for mental fatigue. Each item is scored dichotomously (0 = no, 1 = yes). The physical fatigue score is the sum of items 1 to 8 (maximum score of 8), and the mental fatigue score is the sum of items 9 to 14 (maximum score of 6). The total fatigue score is the sum of both dimensions, with a maximum score of 14. Higher scores reflect greater fatigue levels. Currently, there is no standardized cutoff value for the FS-14 scale, but some studies suggest that a score of 7 or higher can be defined as fatigue [27]. In this study, the Cronbach’s α coefficient for the scale was 0.847.

### 2.3. Statistical Analysis

The general characteristics of the participants were described using frequency and percentages, while the scores for each scale were presented as means and standard deviations (SD). Since all questionnaire items were set as mandatory fields in the electronic survey platform, no missing data were present in the dataset. The differences in stressor scores across groups with different characteristics were assessed using *t*-tests or one-way analysis of variance (ANOVA). The relationships between the scores of each stressor were evaluated using Spearman’s correlation analysis.

We employed the LPA model to identify the stressors patterns of 2695 participants based on the scores of the 7 dimensions of the CSSM scale. LPA is a method of Gaussian finite mixture modeling that utilizes maximum likelihood estimation to identify distinct groups or profiles based on participants’ responses to a set of variables or measures [28]. To ensure comparability between the stressors, the scores of each subscale were standardized. We fitted 1–5-profile LPA models under different variance and covariance conditions and selected the most appropriate model based on the following criteria [29]: (1) lower values of the Akaike Information Criterion (AIC), Bayesian Information Criterion (BIC), and sample-adjusted BIC (aBIC), which reflect better model fit by penalizing model complexity; (2) entropy, with values closer to 1 indicating better distinction between profiles, was considered acceptable when above 0.8; (3) the percentage of the smallest profile, which should exceed 5% to ensure meaningful subgroup sizes and avoid overfitting to rare patterns; (4) the Bootstrapped Likelihood Ratio Test (BLRT), which compares models with k versus k–1 profiles; and (5) the model’s simplicity and theoretical interpretability. Once the optimal model was determined, each participant was assigned to the latent profile based on the posterior probability. The model with varying variances and varying covariances (VVV) was ultimately chosen based on goodness of fit (Figure S1) and interpretability, dividing all participants into two distinct profiles. The larger profile, comprising 92.2% of participants, was designated as the “typical group”, while the smaller profile (7.8%) was labeled as the “atypical group” for this study. Subsequently, general characteristics across different stress pattern groups were compared with Chi-square tests.

Generalized linear models (GLMs) were utilized to evaluate the associations of individual stressors and stressor patterns with the burnout and fatigue scales. Furthermore, the WQS models were applied to investigate the relationships between multiple stressors and the burnout and fatigue scales, as well as to identify the main stressors. WQS is a commonly used mixture analysis method that accounts for both the relationship between the outcome and individual predictors, as well as the correlations among these predictors [29]. In this study, a WQS index was calculated using seven stressor scores, and its association with occupational burnout and fatigue was estimated. In WQS models, each stressor was assigned a weight that reflects its contribution to the overall effect, with the total of all weights summing to 1. Larger weights indicated a stronger relationship with the outcomes. An increase of one unit in the WQS index corresponded to a one-quartile increase in overall stressor scores. The WQS index was estimated using 500 bootstrap samples, with 60% of the data reserved for validation.

The association models, GLM and WQS, were adjusted for the following potential covariates: participants’ age (≤40 or >40), sex (male or female), educational level (bachelor’s degree and below, or graduate degree and above), marital status (single or married), monthly income (≤10,000 or >10,000), hospital classification (primary, secondary, or tertiary), occupational type (physician, nurse, or others), work year (≤20 or >20), daily work time (hours), and weekly night shifts.

All statistical analyses were performed in R (version 4.1.2). The LPA models were conducted using the R package ‘tidyLPA’ (version 1.1.0) [30] and ‘mclust’ (version 6.0.0) [31], while WQS models were implemented with ‘gWQS’ (version 3.0.5) [32]. The statistical significance was considered at a confidence level of 0.05 for a two-tailed test.

## 3. Results

### 3.1. The Characteristics of Participants

As demonstrated in Table 1, a total of 2695 HCWs participated in the survey, with women comprising the majority at 81.0%. Over half of the respondents were younger than 40 years old (66.6%). HCWs from primary, secondary, and tertiary hospitals made up 14.4%, 48.8%, and 36.8% of the sample, respectively. Doctors and nurses accounted for 25.8% and 49.4% of the participants. Compared to their counterparts, HCWs who were male, held graduate degrees or above, worked in tertiary hospitals, or served as physicians reported higher levels of occupational pressure. Those working in surgical departments also experienced greater stress than those in other departments, although the difference compared to HCWs in internal medicine was not statistically significant (Table S2). Additionally, longer daily working hours and increased weekly night shifts significantly elevated occupational stress among HCWs. Moreover, unhealthy lifestyle habits (smoking and drinking) were also associated with increased stress.

### 3.2. Work-Related Stress, Occupational Burnout, and Fatigue

The average score (SD) of the CSSM scale among study participants was 2.88 (0.73), with dimension scores ranging from 2.13 to 3.52. The highest score was observed in the doctor–patient relationship dimension, while the lowest score was in the career development dimension (Table S3). Scores across all dimensions were significantly positively correlated (r: 0.23 to 0.59, *p* < 0.001), as shown in Figure S2.

Among the 1963 participants who completed the MBI-GS scale, the average occupational burnout score (SD) was 2.45 (1.23). A total of 1512 HCWs (77.0%) were categorized as experiencing mild burnout, with 392 (20.0%) classified as experiencing severe burnout. The mean scores (SD) for the three subscales, EE, DP, and PA, were 2.95 (1.69), 2.22 (1.76), and 2.01 (1.55), respectively. EE and DP were strongly positively correlated (r = 0.80, *p* < 0.001), while PA showed a weak negative correlation with EE (r = –0.05, *p* = 0.039). No significant correlation was observed between DP and PA (*p* = 0.525). Among the 732 HCWs who completed the FS-14 scale, the mean total fatigue score (SD) was 8.51 (3.68), with mental fatigue and physical fatigue scores of 2.78 (1.75) and 5.73 (2.48), respectively. These two dimensions were correlated positively (r = 0.50, *p* < 0.001). When using 7 as the cutoff value, 521 (71.2%) of HCWs were classified as experiencing fatigue.

### 3.3. Work-Related Stressor Pattern

**Figure 1:**
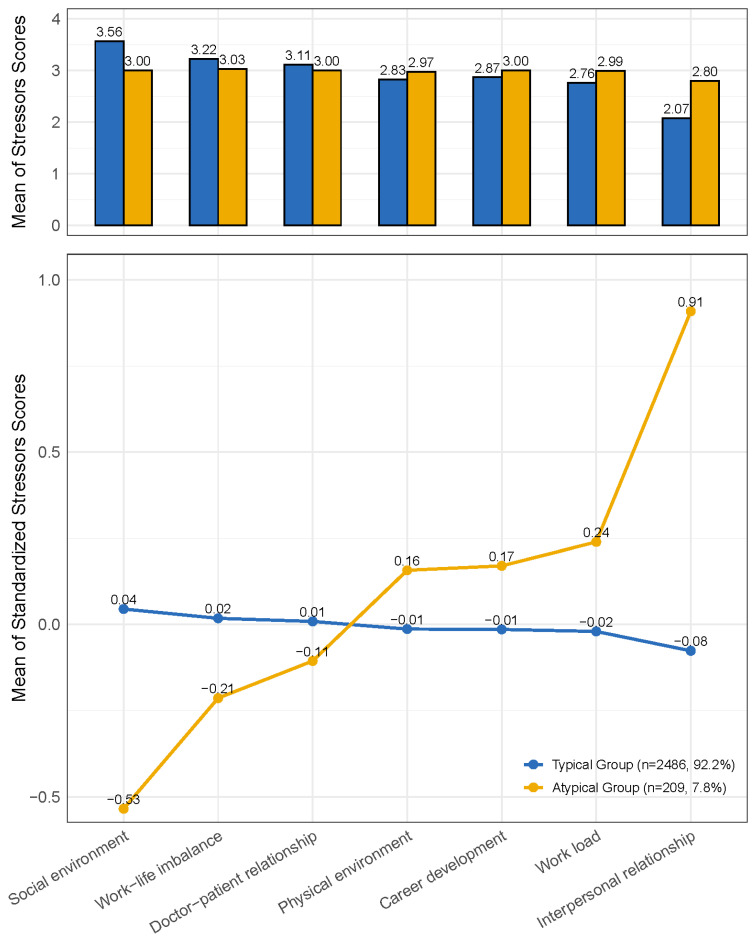
Identification of stressor patterns among participants using the LPA model. Note: “Typical” and “Atypical” refer to the categories representing the majority and minority of HCWs in this study, respectively.

As shown in Figure 1, the LPA model identified two distinct work-related stressor profiles: “typical group” and “atypical group”. A notable characteristic of the “atypical group” is that the raw scores (prior to standardization) for all stressors were closely aligned, suggesting a more evenly distributed level of stress across different sources. Compared to the “typical group”, HCWs in the “atypical group” scored lower on dimensions such as social environment, work–life imbalance, and doctor–patient relationship, while scoring higher on physical environment, career development, workload, and interpersonal relationships.

Further, a Chi-square analysis was conducted to compare the basic demographic and work-related characteristics of HCWs between these two groups. The results revealed that the atypical group had a higher proportion of HCWs with lower education levels, lower monthly incomes, affiliations with primary and secondary hospitals, junior professional titles, and less than 20 years of work experience, compared to the typical group (Figure S3).

### 3.4. The Associations of Occupational Stress with Occupational Burnout and Fatigue

#### 3.4.1. Associations of Individual Stressors with Outcomes

The results of the GLMs demonstrated that all specific occupational stressors were significantly positively associated with both occupational burnout (β: 0.444~0.956, *p* < 0.001) and fatigue (β: 1.384~3.404, *p* < 0.001), as shown in Table 2 and Table 3. For occupational burnout, as well as the two subdimensions, EE and DP, the most influential occupational stressors were career development and work–life imbalance, while for the PA dimension, interpersonal relationships and career development had the strongest effects. Regarding fatigue, both physical and mental, work–life imbalance emerged as the most impactful stressor.

When all occupational stressors were included in the same GLM, career development and work–life imbalance remained the most influential stressors for occupational burnout and fatigue, respectively (Tables S4 and S5). However, some associations were no longer significant, particularly the associations between the physical environment and outcomes, except for their persistent association with EE scores.

#### 3.4.2. Associations of Stressor Patterns with Outcomes

The results of the GLM analysis examining group assignment by the LPA model in relation to occupational burnout and fatigue were presented in Table 4. Compared to the “typical group”, HCWs in the “atypical group” exhibited significantly higher overall burnout scores (β = 0.325, 95% CI: 0.122, 0.528), driven primarily by a notable increase in the PA dimension (β = 1.003, 95% CI: 0.746, 1.259), whereas no significant changes were observed in the other two dimensions (*p* > 0.05). Additionally, no significant associations were found between LPA group assignment and fatigue scores (*p* > 0.05). Given the group size disparity, we conducted additional GLM analysis with robust standard errors. The results remained largely consistent with the original findings (Table S6).

#### 3.4.3. Associations of Combined Stressors with Outcomes

**Figure 2:**
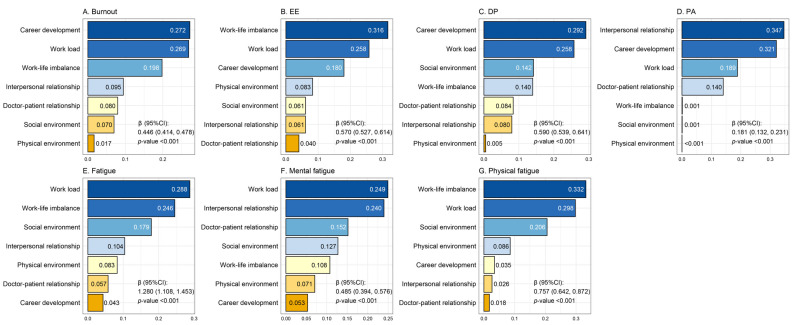
Estimated WQS index weights for occupational burnout (**A**–**D**) and fatigue (**E**–**G**). Models were adjusted for participants’ age, sex, educational level, marital status, monthly income, hospital classification, occupational type, work year, daily work time, and weekly night shifts.

The WQS analysis revealed a significant positive association of the combined exposure to seven occupational stressors with both occupational burnout (β = 0.446, 95% CI: 0.414, 0.478) and fatigue (β = 1.280, 95% CI: 1.108, 1.453). The contributions of each stressor to the associations are illustrated in Figure 2 and Table S7. For occupational burnout, career development, workload, and work–life imbalance emerged as the primary contributors, with weights of 0.272, 0.269, and 0.198, respectively, all exceeding the average weight threshold. For EE and DP, the primary stressors were work–life imbalance (weighted 0.316) and career development (weighted 0.292), respectively, with workload ranking second in both dimensions. In contrast, for PA, the leading stressor was interpersonal relationships (weighted 0.347), indicating a distinct pattern of influence. In the association with fatigue, workload, work–life imbalance, and social environment were identified as the primary drivers, with weights of 0.288, 0.246, and 0.179, respectively. The primary contributors to physical fatigue were similar to those for overall fatigue, while mental fatigue was mainly influenced by workload, interpersonal relationships, and doctor–patient relationship (weighted 0.249, 0.240, and 0.152, respectively).

## 4. Discussion

This study, based on a cross-sectional questionnaire survey, described the status of occupational stress, burnout, and fatigue among HCWs in Shanghai, China. Various statistical models were employed to explore the associations between work-related stress and both occupational burnout and fatigue. The findings revealed a high prevalence of occupational burnout and fatigue among HCWs. All occupational stressors investigated in this study were significantly positively associated with burnout and fatigue. Specifically, career development and workload were identified as the primary contributors to burnout, while workload and work–life imbalance were the main contributors to fatigue. Using the LPA model, two occupational stress patterns were identified. HCWs characterized by higher stress levels in physical environment, career development, workload, and interpersonal relationships exhibited higher burnout scores, particularly in the PA dimension.

Occupational stress remains a longstanding issue in modern healthcare, significantly impacting the well-being of HCWs. This study employed the CSSM scale to evaluate stress across seven dimensions. The findings revealed that, among HCWs, a poor doctor–patient relationship emerged as the highest-rated stressor, likely due to the emotional labor and high expectations associated with patient interactions [33]. Further analysis using the LPA model identified two distinct stress patterns: 92.2% of HCWs were classified into a stress pattern predominantly driven by the social environment, which encompasses negative public opinion and deficiencies in legal regulations and the healthcare insurance system, while the remaining 7.8% exhibited a more balanced distribution of stress across all sources. These results indicated that increasing awareness of healthcare work and enhancing public understanding for the challenges faced by HCWs can effectively reduce misunderstandings and negative comments, thereby lowering occupational stress among HCWs. It is worth noting that although a two-profile solution was statistically optimal, this classification may simplify the complexity of occupational stress experiences among HCWs.

In addition to identifying stress patterns, this study also examined individual characteristics associated with higher stress levels. It was found that male HCWs were more susceptible than their female counterparts, a finding that contrasts with studies conducted in other countries [34,35]. This discrepancy may be influenced by cultural norms and gender roles in China. Previous studies have suggested that Chinese men are culturally expected to bear greater financial and professional responsibilities, and may be less likely to seek emotional support or adopt adaptive coping strategies [36,37]. Consistent with findings from other studies, this study found that lower education levels, longer working hours, and night shifts were associated with higher occupational stress, which may be attributed to an imbalance between workload and compensation or benefits [34,35].

Occupational burnout and fatigue are prevalent challenges faced by HCWs globally, adversely impacting both their physical and mental health, as well as the quality of medical care they deliver. Although occupational burnout and fatigue are not issues exclusive to HCWs, their prevalence and severity were significantly higher in this group compared to other professions [38]. This study identified alarmingly high rates of occupational burnout (77.0%) among HCWs in Shanghai, closely aligning with the findings of a meta-analysis by Zheng et al. [39], which included 64 studies involving over 40,000 physicians in China and reported a burnout prevalence of 75.48%. However, this rate was substantially higher than the 67% burnout prevalence reported by Rotenstein et al. in a meta-analysis of 182 studies spanning 45 countries [40]. It also significantly exceeds the 37.9% burnout rate reported among physicians in the United States [38]. One major reason for the higher burnout rates among Chinese HCWs is the relative shortage of human resources in the healthcare system. By the end of 2019, there were only 6.41 public health professionals per 10,000 population, resulting in a substantial workforce deficit [41]. In addition, Chinese HCWs are subject to high societal expectations and frequently encounter strained doctor–patient relationships, which may further exacerbate psychological stress and burnout [42]. Additionally, the average fatigue score of HCWs in this study was 8.51, exceeding those reported in studies from Liaoning (7.96) and Guangdong (7.29) provinces in China [11,43]. These findings highlighted the severe levels of occupational burnout and fatigue among HCWs in China, with the situation being particularly acute in Shanghai. Factors such as the city’s high population density, centralized medical resources, and intense competition within the healthcare industry further exacerbate these challenges.

The results of association analysis indicated that occupational stress from all sources was significantly associated with both occupational burnout and fatigue. Furthermore, the WQS models identified distinct primary contributing stressors for these outcomes. The dominance of workload as a stressor across nearly all dimensions of burnout and fatigue highlighted the significant strain HCWs experienced, largely driven by persistent service demands compounded by workforce shortages [44]. Additionally, career development was identified as a major contributor to burnout, suggesting that HCWs may face a lack of advancement opportunities, unclear career paths, or inadequate recognition for their work, leading to feelings of frustration and demotivation. This can be especially pronounced in high-pressure environments like healthcare, where the intensity of work often leaves little time for personal or professional growth. Previous studies have also shown a significant positive correlation between career development, job satisfaction, and retention rates, emphasizing the importance of offering HCWs clear career pathways and opportunities for professional growth [45]. Based on these findings, addressing burnout and fatigue among HCWs can begin with managing workload to alleviate excessive strain, enhancing career development opportunities to boost motivation and job satisfaction, and offering clearer career pathways along with better recognition to promote professional growth.

Furthermore, we observed that in the stress pattern characterized by higher interpersonal relationship scores, burnout was significantly elevated, particularly in the PA dimension. Notably, interpersonal relationships were the stressor with the greatest contribution to reduced PA in the WQS model. These consistent findings from two analytical approaches enhance the robustness of our results. The reduction in PA may be partly attributed to a lack of organizational recognition and reward practices, with the quality of interpersonal relationships influencing how such recognition is conveyed and perceived [46,47]. Positive feedback from supervisors and supportive interactions with colleagues can enhance job satisfaction and perceived recognition, whereas poor interpersonal dynamics may obstruct such feedback, leaving HCWs feeling undervalued and demotivated [48]. Moreover, positive relationships may themselves serve as protective buffers against stress by providing emotional support and fostering a collaborative work environment. Conversely, interpersonal conflicts and lack of support can intensify feelings of inefficacy. Thus, enhancing communication, teamwork, and conflict resolution strategies within healthcare settings can be instrumental in mitigating the decline in PA and the occurrence of burnout among HCWs [49,50,51]. However, the lack of a significant association between the LPA-derived stressor profiles and fatigue may be partly explained by the fact that reduced personal accomplishment was not a component of the fatigue measure used in this study.

This study investigated work-related stress from multiple sources, occupational burnout, and fatigue experienced by HCWs in Shanghai, utilizing various statistical models to explore associations and identify key contributors. However, several limitations should be acknowledged. First, the cross-sectional design limited the ability to establish causal relationships, underscoring the need for longitudinal or intervention-based research. Second, the reliance on self-reported questionnaires may introduce a degree of reporting bias. Third, convenience sampling was adopted in the second phase of recruitment due to practical constraints during data collection. While this method facilitated adequate sample size and diversity across roles, we acknowledge that it may introduce selection bias. Likewise, although the study achieved a high effective response rate (99.8%), response bias cannot be ruled out, particularly if individuals experiencing higher stress levels were either more motivated to express their concerns or less likely to participate due to emotional exhaustion. Lastly, as the study focused exclusively on HCWs in Shanghai, caution is warranted when extrapolating the findings to other regions. However, many of the stressors—such as workload, limited career development, and interpersonal challenges—are common across healthcare systems globally. Therefore, the findings may offer insights applicable to international healthcare settings, with appropriate contextual considerations.

## 5. Conclusions

This study revealed a substantial burden of occupational burnout and fatigue among HCWs in Shanghai, China. The excessive workload and limited career development opportunities were key drivers of burnout, while workload and work–life imbalance primarily contributed to fatigue. Interpersonal relationships emerged as a critical factor influencing personal accomplishment. These highlighted systemic workforce challenges that demand urgent attention, and underscored the need for multifaceted interventions including optimized workload allocation, enhanced career development programs, workplace relationship improvement initiatives, and comprehensive mental health support.

Although focused on Shanghai’s healthcare context, these findings provide valuable implications for addressing similar challenges in healthcare systems worldwide, particularly in high-pressure urban environments facing workforce sustainability issues. Further studies would benefit from longitudinal or experimental designs to verify causal pathways and systematically evaluate intervention outcomes.

## Figures and Tables

**Table 1:** Detailed characteristics of the study sample (*n*= 2695).

Variables	Frequency (Percentage) *n* (%)	CSSM Scores Mean ± SD	*t*/*F*	*p*-Value
General characteristics				
Sex				
Male	512 (19.0%)	3.05 ± 0.58	−5.61	<0.001
Female	2183 (81.0%)	2.89 ± 0.60		
Age (year)				
≤40	1796 (66.6%)	2.91 ± 0.61	−0.99	0.324
>40	899 (33.4%)	2.94 ± 0.58		
Education level				
Junior college degree or below	734 (27.2%)	2.86 ± 0.59	24.35	<0.001
Bachelor’s degree	1599 (59.3%)	2.91 ± 0.61		
Graduate degree and above	362 (13.4%)	3.12 ± 0.53		
Marital status				
Married	2009 (74.8%)	2.92 ± 0.60	0.31	0.735
Single	686 (25.5%)	2.94 ± 0.63		
Monthly income (RMB)				
≤10,000	1470 (54.5%)	2.93 ± 0.59	0.99	0.324
>10,000	1225 (45.5%)	2.91 ± 0.61		
Work-related features				
Hospital classification				
Primary hospital	388 (14.4%)	2.83 ± 0.56	21.98	<0.001
Secondary hospital	1315 (48.8%)	2.88 ± 0.59		
Tertiary hospital	992 (36.8%)	3.02 ± 0.63		
Department				
Internal medicine	480 (17.8%)	2.95 ± 0.58	10.30	<0.001
Surgery	356 (13.2%)	3.04 ± 0.63		
Others	1859 (69.0%)	2.89 ± 0.60		
Occupational type				
Physician	695 (25.8%)	3.11 ± 0.52	56.84	<0.001
Nurse	1332 (49.4%)	2.89 ± 0.63		
Others	668 (24.8%)	2.78 ± 0.58		
Professional title				
Junior	1427 (52.9%)	2.90 ± 0.62	−1.92	0.055
Intermediate and senior	1268 (47.1%)	2.95 ± 0.58		
Work year				
<5	587 (21.8%)	2.88 ± 0.63	1.95	0.142
5~20	1326 (49.2%)	2.93 ± 0.61		
>20	782 (29.0%)	2.93 ± 0.58		
Daily work time (hours)				
<8	479 (17.8%)	2.72 ± 0.58	72.97	<0.001
8~10	1933 (71.7%)	2.92 ± 0.59		
>10	283 (10.5%)	3.25 ± 0.55		
Weekly night shifts				
None	1293 (48.0%)	2.80 ± 0.57	66.88	<0.001
1~5	892 (33.1%)	2.96 ± 0.61		
≥10	510 (18.9%)	3.15 ± 0.60		
Lifestyle factors				
Drink				
No	2466 (91.5%)	2.91 ± 0.61	−4.25	<0.001
Yes	229 (8.5%)	3.07 ± 0.56		
Smoke				
No	2575 (95.5%)	2.91 ± 0.60	−3.49	<0.001
Yes	120 (4.5%)	3.12 ± 0.63		
Physical activity				
No	2037 (75.6%)	2.92 ± 0.60	0.02	0.980
Yes	658 (24.4%)	2.92 ± 0.61		

**Table 2:** Associations between occupational stress and occupational burnout for HCWs by GLMs (*n* = 1963).

Occupational Stress	Burnout		EE		DP		PA	
	β (95% CI)	*p*-Value	β (95% CI)	*p*-Value	β (95% CI)	*p*-Value	β (95% CI)	*p*-Value
Career development	0.956 (0.883, 1.030)	<0.001	1.143 (1.037, 1.250)	<0.001	1.204 (1.092, 1.315)	<0.001	0.460 (0.348, 0.571)	<0.001
Interpersonal relationships	0.715 (0.632, 0.797)	<0.001	0.787 (0.669, 0.905)	<0.001	0.872 (0.749, 0.994)	<0.001	0.461 (0.348, 0.574)	<0.001
Work–life imbalance	0.747 (0.681, 0.814)	<0.001	1.103 (1.014, 1.192)	<0.001	0.914 (0.813, 1.014)	<0.001	0.106 (0.008, 0.204)	0.034
Physical environment	0.620 (0.555, 0.686)	<0.001	0.862 (0.772, 0.952)	<0.001	0.786 (0.689, 0.884)	<0.001	0.132 (0.039, 0.225)	0.006
Doctor–patient relationship	0.444 (0.379, 0.510)	<0.001	0.580 (0.489, 0.671)	<0.001	0.594 (0.499, 0.689)	<0.001	0.114 (0.027, 0.202)	0.011
Social environment	0.546 (0.486, 0.606)	<0.001	0.732 (0.649, 0.815)	<0.001	0.745 (0.657, 0.833)	<0.001	0.098 (0.015, 0.182)	0.021
Workload	0.670 (0.603, 0.737)	<0.001	0.874 (0.781, 0.968)	<0.001	0.848 (0.748, 0.949)	<0.001	0.218 (0.123, 0.313)	<0.001

Note: The GLMs were adjusted for participants’ age, sex, educational level, marital status, monthly income, hospital classification, occupational type, work year, daily work time, and weekly night shifts.

**Table 3:** Associations between occupational stress and fatigue for HCWs by GLMs (*n* = 732).

Occupational Stress	Fatigue		Mental Fatigue		Physical Fatigue	
	β (95% CI)	*p*-Value	β (95% CI)	*p*-Value	β (95% CI)	*p*-Value
Career development	2.032 (1.189, 2.875)	<0.001	0.935 (0.520, 1.349)	<0.001	1.098 (0.523, 1.672)	<0.001
Interpersonal relationships	1.715 (0.922, 2.508)	<0.001	0.841 (0.462, 1.221)	<0.001	0.873 (0.330, 1.417)	0.002
Work–life imbalance	3.404 (2.603, 4.205)	<0.001	1.378 (0.974, 1.782)	<0.001	2.026 (1.480, 2.573)	<0.001
Physical environment	1.728 (1.024, 2.432)	<0.001	0.629 (0.284, 0.975)	<0.001	1.099 (0.623, 1.575)	<0.001
Doctor–patient relationship	1.384 (0.716, 2.052)	<0.001	0.649 (0.326, 0.972)	<0.001	0.735 (0.279, 1.191)	0.002
Social environment	2.024 (1.430, 2.618)	<0.001	0.640 (0.344, 0.937)	<0.001	1.384 (0.984, 1.783)	<0.001
Workload	2.086 (1.469, 2.703)	<0.001	0.811 (0.505, 1.118)	<0.001	1.275 (0.855, 1.695)	<0.001

Note: The GLMs were adjusted for participants’ age, sex, educational level, marital status, monthly income, hospital classification, occupational type, work year, daily work time, and weekly night shifts.

**Table 4:** Associations of group assignment by LPA model with occupational burnout and fatigue.

	β (95% CI)	*p*-Value
Burnout	0.325 (0.122, 0.528)	0.002
EE	−0.034 (−0.314, 0.247)	0.814
DP	0.126 (−0.168, 0.420)	0.402
PA	1.003 (0.746, 1.259)	<0.001
Fatigue	−0.194 (−1.151, 0.763)	0.691
Mental fatigue	−0.222 (−0.677, 0.233)	0.338
Physical fatigue	0.028 (−0.618, 0.675)	0.932

Note: The “typical group” served as the reference category. The GLMs were adjusted for participants’ age, sex, educational level, marital status, monthly income, hospital classification, occupational type, work year, daily work time, and weekly night shifts.

## Data Availability

The raw data supporting the conclusions of this article will be made available by the authors on request.

## Supplementary Materials

The following supporting information can be downloaded at: https://www.mdpi.com/article/10.3390/healthcare13131600/s1, Figure S1: Fit indices for 1–5 profile LPA models under four parametrization conditions. Figure S2: Correlation matrix of occupational stress scores across CSSM dimensions. Figure S3: Comparisons of the demographic and work-related characteristics between the two groups assigned by the LPA model. Table S1: Psychometric properties of the CSSM scale in this study. Table S2: Post hoc comparisons of occupational stress scores across subgroups with significant differences in ANOVA results. Table S3: The total and subscale scores of the CSSM, MBI-GS, and FS-14. Table S4: Associations between occupational stress and occupational burnout for HCWs by GLMs including all stressors (n = 1963). Table S5: Associations between occupational stress and fatigue for HCWs by GLMs including all stressors (n = 732). Table S6: Associations of group assignment by LPA model with occupational burnout and fatigue corrected with robust standard errors. Table S7: Estimated WQS index weights for occupational burnout and fatigue.
