# Supplementary material for: Occupational Stress, Burnout, and Fatigue Among Healthcare Workers in Shanghai, China: A Questionnaire-Based Cross-Sectional Survey
Source: Healthcare (Basel). 2025 Jul 3;13(13):1600. doi: 10.3390/healthcare13131600 (PMC12250260; doi:10.3390/healthcare13131600)
Supplement: Supplementary file 1 [file healthcare-13-01600-s001.zip › healthcare-3585990-supplementary.pdf]

Supplementary Materials

Occupational Stress, Burnout, and Fatigue Among Healthcare Workers in Shanghai, China: A Questionnaire-Based Cross-Sectional Survey

Author names and affiliations:

Qiaochu Wang [1,2], Jiayun Ding [1,2], Yiming Dai [1,2], Sijia Yang [3,*] and Zhijun Zhou [1,2,*]

[1] Key Laboratory of Public Health Safety of Ministry of Education, School of Public Health, Fudan Uni-versity, No. 130 Dong'an Road, Shanghai 200032, China; 21111020010@m.fudan.edu.cn (Q.W.); 22211020077@m.fudan.edu.cn (J.D.); 20111020031@fudan.edu.cn (Y.D.)

[2] Key Laboratory of Health Technology Assessment of National Health Commission, School of Public Health, Fudan University, No. 138 Yixueyuan Road, Shanghai 200032, China;

[3] Shanghai Municipal Center for Disease Control and Prevention, No. 1399 Shen Hong Road, Minhang District, Shanghai 200336, China

[*] Correspondence: yangsijia@scdc.sh.cn (S.Y.); zjzhou@fudan.edu.cn (Z.Z.)

Contents

Table S1. Psychometric properties of the CSSM scale in this study.

Figure S1. Fit indices for 1-5 profile LPA models under four parametrization conditions.

Table S2. Post hoc comparisons of occupational stress scores across subgroups with significant differences in ANOVA results.

Table S3. The total and subscale scores of the CSSM, MBI-GS, and FS-14.

Figure S2. Correlation matrix of occupational stress scores across CSSM dimensions.

Figure S3. Comparisons of the demographic and work-related characteristics between the two groups assigned by LPA model.

Table S4. Associations between occupational stress and occupational burnout for HCWs by GLMs including all stressors (*n*=1963).

Table S5. Associations between occupational stress and fatigue for HCWs by GLMs including all stressors (*n*=732).

Table S6. Associations of group assignment by LPA model with occupational burnout and fatigue corrected with robust standard errors.

Table S7. Estimated WQS index weights for occupational burnout and fatigue.

Table S1. Psychometric properties of the CSSM scale in this study.

Dimension/Item	Cronbach's α	Item-total correlation	CFA loading
Career development	0.902	-	-
Item 1. My department has an unreasonable bonus distribution system.	-	0.764	0.766
Item 2. My department lacks an incentive mechanism.	-	0.776	0.779
Item 3. The professional title evaluation and appointment system in my institution is unfair.	-	0.790	0.796
Item 4. Internal competition is intense, and my chances of promotion are slim.	-	0.733	0.730
Item 5. My supervisor frequently encourages me. (R)	-	0.623	0.609
Item 6. My work performance is rarely recognized.	-	0.683	0.672
Item 7. I see no promising career prospects.	-	0.770	0.774
Item 8. I am dissatisfied with my current professional title and position.	-	0.553	0.546
Item 9. I have very few opportunities for further training or external exchanges.	-	0.713	0.723
Interpersonal relationships	0.844	-	-
Item 10. There are frequent conflicts and tensions among colleagues.	-	0.763	0.766
Item 11. I experience bullying in the workplace.	-	0.821	0.838
Item 12. There is a lack of mutual respect among coworkers.	-	0.830	0.861
Item 13. I struggle to maintain relationships with certain colleagues.	-	0.647	0.640
Item 14. I encounter harassment at work, including hostile language and behavior.	-	0.554	0.559
Work-life imbalance	0.823	-	-
Item 15. My work leaves me no time to spend with my family.	-	0.834	0.861
Item 16. My job doesn't allow me time to pursue personal hobbies and interests.	-	0.853	0.899
Item 17. I don't have time to take vacations.	-	0.771	0.779
Item 18. I can decide when to take breaks. (R)	-	0.483	0.443
Item 19. Frequent shift rotations/night shifts. (R)	-	0.542	0.519
Physical environment	0.827	-	-
Item 20. My work environment carries a risk of infectious diseases.	-	0.640	0.678
Item 21. There are consistently unpleasant odors in my workplace.	-	0.783	0.810
Item 22. Toxic chemicals are present in my work environment.	-	0.774	0.771
Item 23. I am exposed to radiation in my workplace.	-	0.695	0.704
Doctor-patient relationship	0.861	-	-
Item 24. Patients or their family members often make unreasonable demands.	-	0.613	0.620
Item 25. Dealing with violent patients or family members is distressing to me.	-	0.908	0.927
Item 26. Coping with verbal abuse from patients and their families is troubling.	-	0.905	0.934
Social environment	0.876	-	-
Item 27. There is excessive negative media coverage targeting healthcare professionals.	-	0.729	0.753
Item 28. Current laws and regulations inadequately protect medical practitioners.	-	0.863	0.877
Item 29. The healthcare insurance system remains insufficient to address medical risks.	-	0.862	0.896
Workload	0.756	-	-
Item 30. I have work tasks that cannot be completed by the deadline.	-	0.733	0.773
Item 31. Due to excessive workload, I have to neglect certain job responsibilities.	-	0.738	0.790
Item 32. I spend excessive time handling medical records and documentation.	-	0.573	0.601

Note: Items marked with "(R)" were reverse-scored.

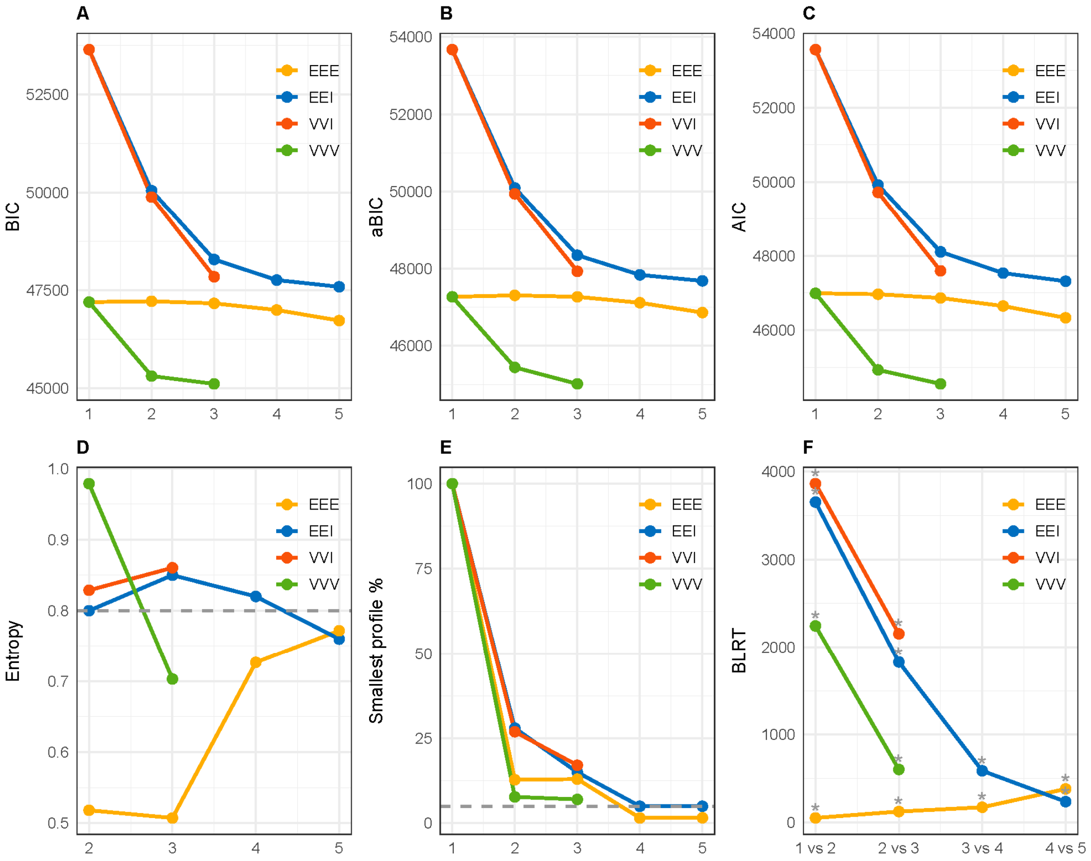

Figure S1. Fit indices for 1-5 profile LPA models under four parametrization conditions, including (A) BIC, (B) aBIC, (C) AIC, (D) Entropy, (E) percentage of smallest profile and (F) BLRT results.

Notes: EEE, Equal variances and equal covariances; EEI, Equal variances, and covariances fixed to 0; VVI, Varying variances and covariances fixed to 0; VVV, Varying variances and covariances fixed to 0.

The VVI and VVV models failed to converge when the number of profiles was set to 4 or 5.

Table S2. Post hoc comparisons of occupational stress scores across subgroups with significant differences in ANOVA results.

**Comparison**	**Mean difference (95%CI)**	***p*-value adjusted**
Education level		
Bachelor's degree vs. Junior college degree or below	0.05 (-0.01, 0.11)	0.123
Graduate degree and above vs. Junior college degree or below	0.26 (0.17, 0.35)	<0.001
Graduate degree and above vs. Bachelor's degree	0.21 (0.13, 0.29)	<0.001
Hospital classification		
Secondary vs. Primary	0.05 (-0.03, 0.13)	0.316
Tertiary vs. Primary	0.19 (0.11, 0.28)	<0.001
Tertiary vs. Secondary	0.14 (0.08, 0.20)	<0.001
Department		
Surgery vs. Internal medicine	0.10 (0.00, 0.19)	0.059
Others vs. Internal medicine	-0.06 (-0.13, 0.01)	0.149
Others vs. Surgery	-0.15 (-0.23, -0.07)	<0.001
Occupational type		
Nurse vs. Physician	-0.22 (-0.28, -0.15)	<0.001
Others vs. Physician	-0.33 (-0.41, -0.26)	<0.001
Others vs. Nurse	-0.12 (-0.18, -0.05)	<0.001
Daily work time (hours)		
8~10 vs. <8	0.20 (0.13, 0.28)	<0.001
>10 vs. <8	0.53 (0.43, 0.64)	<0.001
>10 vs. 8~10	0.33 (0.24, 0.41)	<0.001
Weekly night shifts		
1~5 vs. None	0.15 (0.09, 0.21)	<0.001
≥10 vs. None	0.35 (0.28, 0.42)	<0.001
≥10 vs. 1~5	0.19 (0.12, 0.27)	<0.001

Note: Post hoc comparisons were conducted using Tukey's HSD test for variables showing significant group differences in one-way ANOVA.

Table S3. The total and subscale scores of the CSSM, MBI-GS, and FS-14.

Variables	Mean±SD
CSSM (*n*=2695)	
Occupational stress	2.88 ± 0.73
Career development	2.13 ± 0.73
Interpersonal relationships	3.21 ± 0.83
Work-life imbalance	2.84 ± 0.87
Physical environment	3.10 ± 0.92
Doctor-patient relationship	3.52 ± 0.97
Social environment	2.78 ± 0.88
Workload	2.92 ± 0.60
MBI-GS (*n*=1963)	
Occupational burnout	2.45 ± 1.23
Emotional exhaustion (EE)	2.95 ± 1.69
Depersonalization (DP)	2.22 ± 1.76
Reduced personal accomplishment (PA)	2.01 ± 1.55
FS-14 (*n*=732)	
Fatigue	8.51 ± 3.68
Mental fatigue	2.78 ± 1.75
Physical fatigue	5.73 ± 2.48

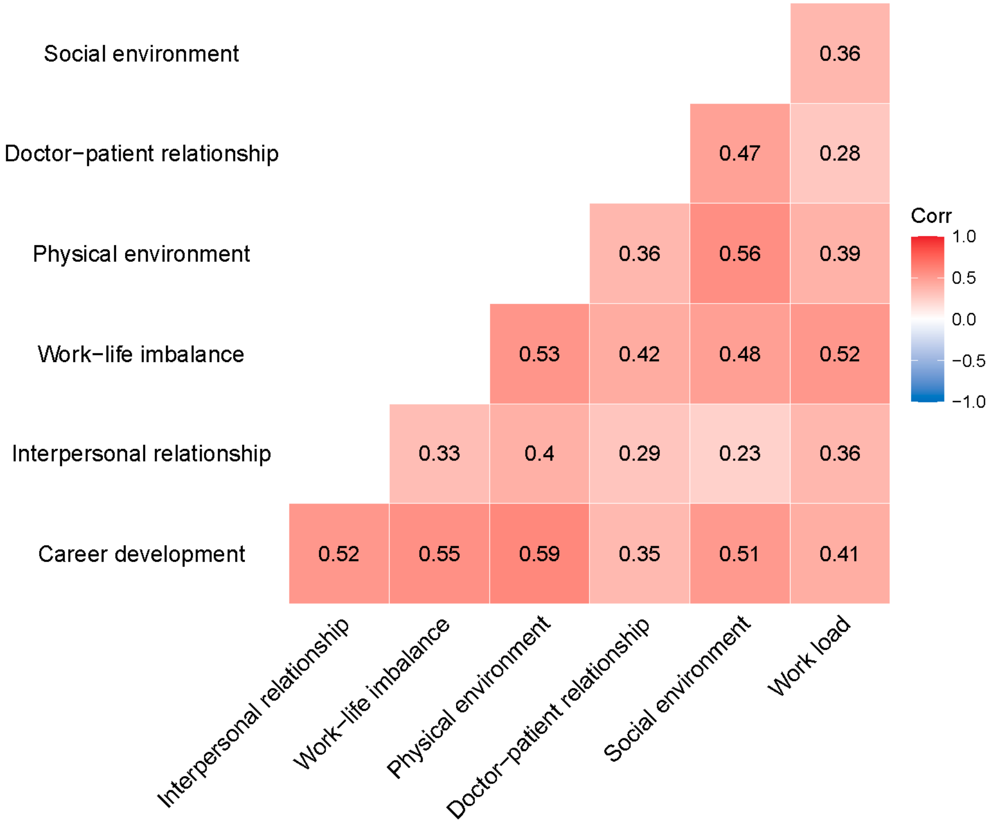

Figure S2. Correlation matrix of occupational stress scores across CSSM dimensions.

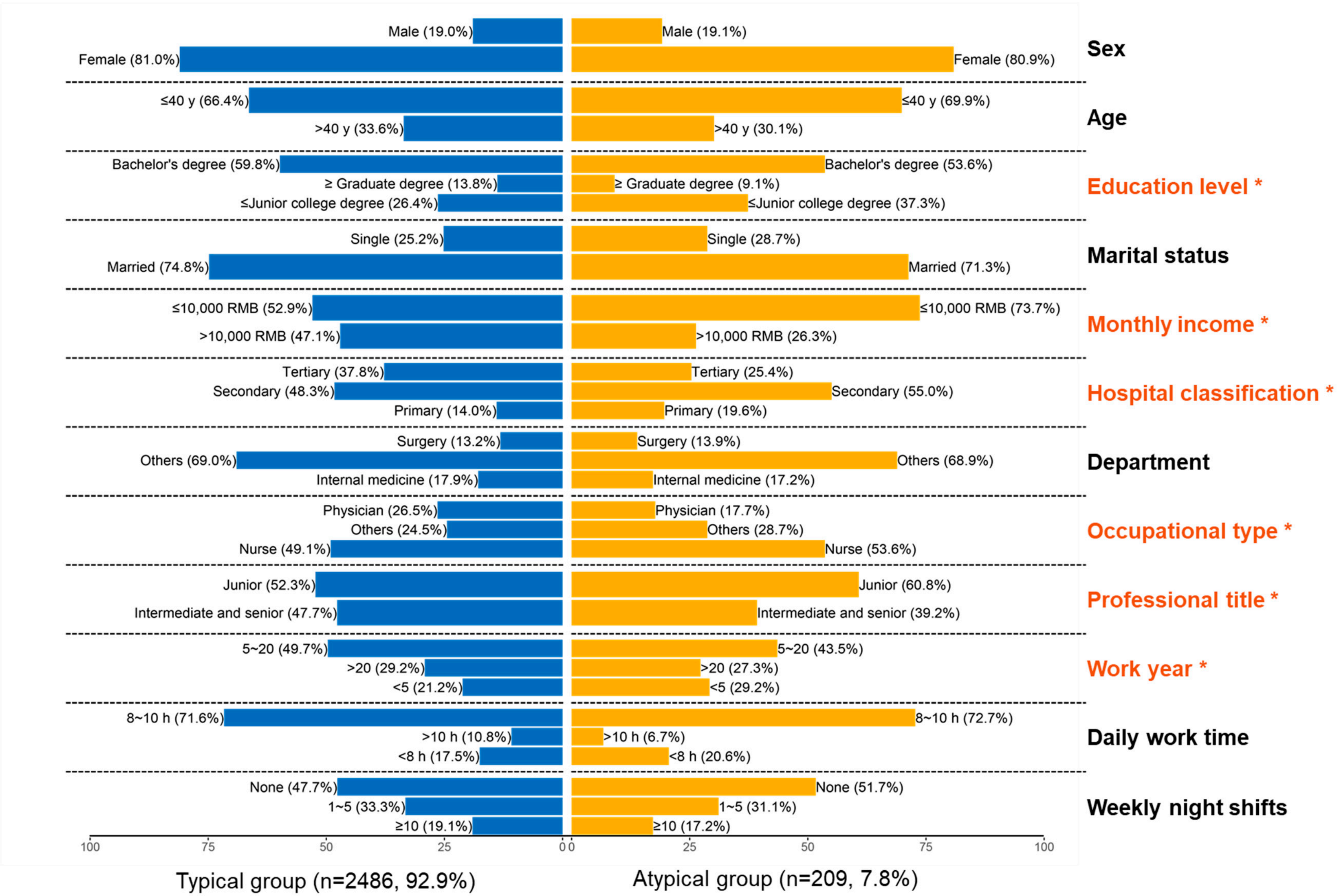

Figure S3. Comparisons of the demographic and work-related characteristics between the two groups assigned by LPA model.

Note: *Indicates statistical significance based on the chi-square test ($p < 0.05$).

Table S4. Associations between occupational stress and occupational burnout for HCWs by GLMs including all stressors (n=1963).

**Occupational stress**	**Burnout**		**EE**		**DP**		**PA**	
	**β (95%CI)**	***p*-value**	**β (95%CI)**	***p*-value**	**β (95%CI)**	***p*-value**	**β (95%CI)**	***p*-value**
Career development	0.449 (0.365, 0.534)	<0.001	0.377 (0.258, 0.496)	<0.001	0.600 (0.468, 0.732)	<0.001	0.396 (0.259, 0.533)	<0.001
Interpersonal relationships	0.191 (0.122, 0.260)	<0.001	0.117 (0.020, 0.214)	0.019	0.217 (0.109, 0.325)	<0.001	0.264 (0.152, 0.376)	<0.001
Work-life imbalance	0.266 (0.197, 0.334)	<0.001	0.598 (0.502, 0.695)	<0.001	0.254 (0.146, 0.361)	<0.001	-0.166 (-0.278, -0.054)	0.004
Physical environment	-0.003 (-0.073, 0.067)	0.932	0.105 (0.006, 0.205)	0.038	-0.049 (-0.159, 0.061)	0.384	-0.102 (-0.217, 0.013)	0.082
Doctor-patient relationship	0.047 (-0.005, 0.099)	0.076	0.034 (-0.039, 0.108)	0.363	0.103 (0.021, 0.184)	0.013	0.009 (-0.076, 0.094)	0.835
Social environment	0.112 (0.052, 0.171)	<0.001	0.145 (0.061, 0.230)	0.001	0.232 (0.139, 0.326)	<0.001	-0.054 (-0.151, 0.043)	0.278
Workload	0.263 (0.205, 0.321)	<0.001	0.323 (0.240, 0.405)	<0.001	0.326 (0.234, 0.417)	<0.001	0.121 (0.026, 0.216)	0.013

Note: The GLMs were adjusted for participants' age, sex, educational level, marital status, monthly income, hospital classification, occupational type, work year, daily work time and weekly night shifts.

Table S5. Associations between occupational stress and fatigue for HCWs by GLMs including all stressors (n=732).

**Occupational stress**	**Fatigue**		**Mental fatigue**		**Physical fatigue**	
	**β (95%CI)**	***p*-value**	**β (95%CI)**	***p*-value**	**β (95%CI)**	***p*-value**
Career development	0.498 (0.056, 0.940)	0.028	0.227 (-0.000, 0.455)	0.051	0.270 (-0.034, 0.575)	0.082
Interpersonal relationships	0.404 (0.042, 0.766)	0.029	0.343 (0.157, 0.529)	<0.001	0.061 (-0.188, 0.310)	0.631
Work-life imbalance	1.080 (0.678, 1.482)	<0.001	0.327 (0.120, 0.534)	0.002	0.753 (0.477, 1.030)	<0.001
Physical environment	0.016 (-0.325, 0.357)	0.926	-0.041 (-0.217, 0.135)	0.646	0.057 (-0.178, 0.292)	0.633
Doctor-patient relationship	0.083 (-0.231, 0.397)	0.606	0.177 (0.015, 0.339)	0.032	-0.094 (-0.310, 0.122)	0.392
Social environment	0.699 (0.393, 1.006)	<0.001	0.149 (-0.009, 0.307)	0.065	0.551 (0.339, 0.762)	<0.001
Workload	0.864 (0.544, 1.184)	<0.001	0.271 (0.106, 0.435)	0.001	0.594 (0.374, 0.814)	<0.001

Note: The GLMs were adjusted for participants' age, sex, educational level, marital status, monthly income, hospital classification, occupational type, work year, daily work time and weekly night shifts.

Table S6. Associations of group assignment by LPA model with occupational burnout and fatigue corrected with robust standard errors.

	**β (95%CI)**	***p*-value**
Burnout	0.325 (0.158, 0.492)	<0.001
EE	-0.034 (-0.308, 0.240)	0.810
DP	0.126 (-0.157, 0.408)	0.383
PA	1.003 (0.716, 1.289)	<0.001
Fatigue	-0.194 (-1.163, 0.775)	0.695
Mental fatigue	-0.222 (-0.633, 0.189)	0.289
Physical fatigue	0.028 (-0.684, 0.741)	0.938

Table S7. Estimated WQS index weights for occupational burnout and fatigue.

**Occupational stressors**	**Burnout**	**EE**	**DP**	**PA**	**Fatigue**	**Mental fatigue**	**Physical fatigue**
Career development	**0.272**	**0.180**	**0.292**	**0.321**	0.043	0.053	0.035
Doctor-patient relationship	0.080	0.040	0.084	0.140	0.057	**0.152**	0.018
Physical environment	0.017	0.083	0.005	0.000	0.083	0.071	0.086
Interpersonal relationship	0.095	0.061	0.080	**0.347**	0.104	**0.240**	0.026
Social environment	0.070	0.061	0.142	0.001	**0.179**	0.127	**0.206**
Work load	**0.269**	**0.258**	**0.258**	**0.189**	**0.288**	**0.249**	**0.298**
Work-life imbalance	**0.198**	**0.316**	0.140	0.001	**0.246**	0.108	**0.332**

Notes: WQS models were adjusted for participants' age, sex, educational level, marital status, monthly income, hospital classification, occupational type, work year, daily work time and weekly night shifts. **Bold** indicates weights greater than the average weight (1/7).
